# Macrophage interferon regulatory factor 4 deletion ameliorates aristolochic acid nephropathy via reduced migration and increased apoptosis

**DOI:** 10.1172/jci.insight.150723

**Published:** 2022-02-22

**Authors:** Kensuke Sasaki, Andrew S. Terker, Jiaqi Tang, Shirong Cao, Juan Pablo Arroyo, Aolei Niu, Suwan Wang, Xiaofeng Fan, Yahua Zhang, Stephanie R. Bennett, Ming-zhi Zhang, Raymond C. Harris

**Affiliations:** 1 Division of Nephrology, Department of Medicine, Vanderbilt University Medical Center, Nashville, Tennessee, USA.; 2 Vanderbilt Center for Kidney Disease, Nashville, Tennessee, USA.; 3 Department of Veterans Affairs, Tennessee Valley Healthcare System, Nashville, Tennessee, USA.

**Keywords:** Nephrology, Chronic kidney disease, Macrophages

## Abstract

Aristolochic acid (AA) is the causative nephrotoxic alkaloid in AA nephropathy, which results in a tubulointerstitial fibrosis. AA causes direct proximal tubule damage as well as an influx of macrophages, although the role of macrophages in pathogenesis is poorly understood. Here, we demonstrate that AA directly stimulates migration, inflammation, and ROS production in macrophages ex vivo. Cells lacking interferon regulatory factor 4 (IRF4), a known regulator of macrophage migration and phenotype, had a reduced migratory response, though effects on ROS production and inflammation were preserved or increased relative to WT cells. Macrophage-specific IRF4-knockout mice were protected from both acute and chronic kidney effects of AA administration based on functional and histological analysis. Renal macrophages from kidneys of AA-treated macrophage-specific IRF4-knockout mice demonstrated increased apoptosis and ROS production compared with WT controls, indicating that AA directly polarizes macrophages to a promigratory and proinflammatory phenotype. However, knockout mice had reduced renal macrophage abundance following AA administration. While macrophages lacking IRF4 can adopt a proinflammatory phenotype upon AA exposure, their inability to migrate to the kidney and increased rates of apoptosis upon infiltration provide protection from AA in vivo. These results provide evidence of direct AA effects on macrophages in AA nephropathy and add to the growing body of evidence that supports a key role of IRF4 in modulating macrophage function in kidney injury.

## Introduction

Aristolochic acid (AA) is the causative nephrotoxic alkaloid in AA nephropathy (AAN). AAN has been most frequently encountered as a cause of kidney disease in the Balkans (1), where AA can be found as a soil contaminant. AA exposures also result from its use in weight loss supplements and traditional medicines in Europe, Asia, and throughout the world (2, 3). AAN presents clinically as a progressive interstitial fibrosis, with decline in renal function, and leads to end-stage kidney disease in patients with repeated exposure. While not commonly encountered by nephrologists in the United States, AAN is the cause of end-stage renal disease in up to 10% of the dialysis population in some endemic areas (4). The toxic effects of AA on renal tubules are linked to epithelial cell G_2_/M arrest, followed by an apoptotic and profibrotic response (5). While these direct effects on renal epithelial cells are well documented, the roles of other cell types involved in the injury process remain largely unexplored in AAN, preventing a complete understanding of its pathogenesis.

Macrophages have been shown to be critical in both the propagation and resolution phases of kidney injury (6). Genetic or pharmacologic manipulation of macrophage function in several models reveals that inhibition or stimulation of macrophage function can ameliorate or exacerbate kidney injury, respectively. Additionally, multiple aspects of macrophage function, including migration ability and inflammatory phenotype, have been well-characterized and correlate with extent of kidney injury. These important findings have been demonstrated in a range of kidney injury models, including ischemia/reperfusion injury (IRI), unilateral ureteral obstruction (UUO), and models of direct proximal tubule injury, but it has not been thoroughly explored in AAN.

Interferon regulatory factor 4 (IRF4) is a member of the IRF family of transcription factors. IRF4 has a role in mediating the macrophage response to Toll-like receptor 4 stimulation, in addition to affecting macrophage polarization and migration. Our group recently reported that deletion of macrophage IRF4 reduces monocyte recruitment following acute kidney injury, leading to decreased injury in both IRI and UUO models (7). Here, we examined how selective macrophage deletion of IRF4 affected kidney injury in a model of AAN.

Our overall goal in this study was to determine the role of macrophages in a model of AAN. We tested the hypothesis that AA directly stimulates macrophages, inducing a migratory and proinflammatory phenotype to potentiate AA-induced kidney injury, and that these effects would be reduced in mice with macrophage-specific deletion of IRF4. We show that AA directly stimulated macrophages in culture to induce migration, apoptosis, ROS production, and inflammation. Effects on migration were prevented in cells lacking IRF4, though effects on ROS production and inflammation were preserved or increased relative to control cells. In vivo, macrophage-specific IRF4 deletion protected from AA-induced kidney injury, inflammation, and fibrosis. Our findings provide mechanistic insight into the role of macrophages in the pathogenesis of AAN, and the protective effect of IRF4 deletion adds to the growing body of evidence that supports a key role of IRF4 in modulating macrophage function in kidney injury.

## Results

**Figure 1:**
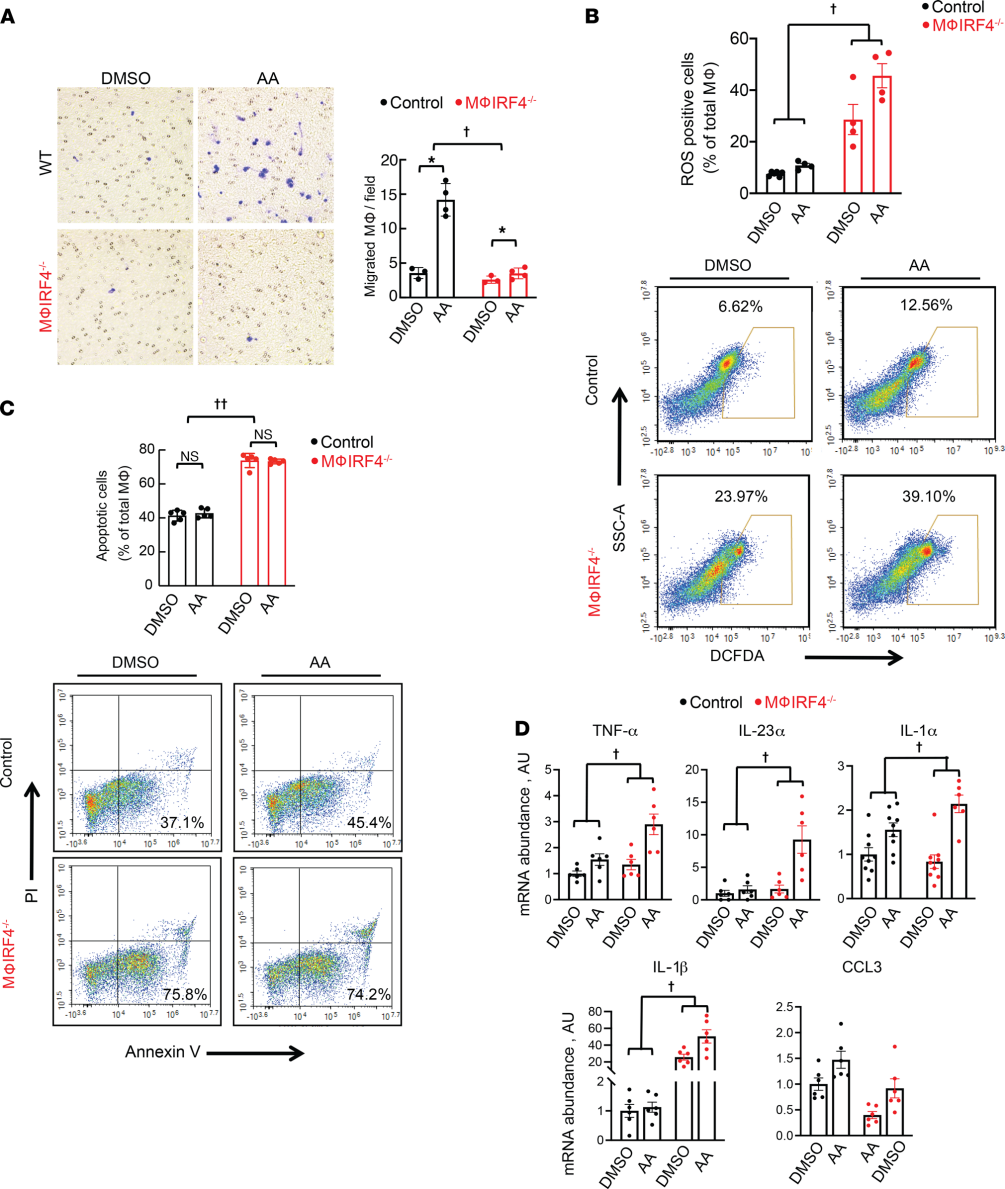
Effects of 3.5 μM AA on peritoneal macrophage inflammatory and migratory phenotype ex vivo. (**A**) Effects of AA culture for 16 hours on WT control and macrophage IRF4^–/–^ macrophage migration ex vivo. Blue indicates positivity by crystal violet stain. (**B**) Effects of AA culture for 16 hours on ROS production in WT control and macrophage IRF4^–/–^ macrophages ex vivo. (**C**) Effects of AA culture for 5 hours on apoptosis in WT control and macrophage IRF4^–/–^ macrophages ex vivo. (**D**) Effects of AA on mRNA abundance of proinflammatory cytokines, including TNF-α, IL-23α, IL-1α, IL-1β, and CCL3, in control and macrophage IRF4^–/–^ macrophages ex vivo. For **A**, *n* = 3 for DMSO groups and 4 for AA groups; *n* = 4 for all groups in **B**, except DMSO-treated control cells, for which *n* = 6. For **C**, *n* = 5 for all groups; *n* = 6 per group in **D**, except for IL-1α, which has *n* = 9 for WT DMSO, WT AA, and knockout DMSO groups and *n* = 6 for knockout AA. †*P* < 0.05 for interaction between genotype and treatment by 2-way ANOVA. ††*P* < 0.05 for genotype effect by 2-way ANOVA. *P < 0.05 by 2-way ANOVA followed by Šidák post hoc test. AA, aristolochic acid; Mϕ, macrophage.

It is known that AA causes direct tubular injury to proximal tubule cells. We hypothesized that AA might also directly stimulate macrophages. To test this, we isolated peritoneal macrophages from WT animals and treated them with AA in culture for 16 hours. As shown in Figure 1, AA led to increased macrophage migration and ROS production (Figure 1, A and B). Shorter treatment, for 5 hours, had no effect on apoptosis (Figure 1C). Transcript abundance of proinflammatory cytokines, including TNF-α, IL-23α, IL-1α, IL-1β, and C-C motif chemokine 3 (CCL3), were all increased following AA exposure (Supplemental Figure 1A; supplemental material available online with this article; https://doi.org/10.1172/jci.insight.150723DS1). Similar increases in cytokine transcript abundance were observed in the macrophage cell line, RAW 264.7, following AA exposure (Supplemental Figure 1B).

To determine the potential role of IRF4 in the AA response, we treated macrophages isolated from macrophage-specific IRF4-knockout animals (macrophage IRF4^–/–^) with AA (Supplemental Figure 2). Macrophage IRF4^–/–^ cells did not respond to AA with increased migration but had increased ROS production compared with WT control cells (Figure 1, A and B, and Supplemental Figure 3). Similar to observations in WT cells, AA itself did not affect cell death, but IRF4 deletion did increase rates of apoptosis following both DMSO and AA treatment (Figure 1C and Supplemental Figure 3). Furthermore macrophage IRF4^–/–^ cells also responded to AA with an increase in transcript abundance of TNF-α, IL-23α, IL-1α, IL-1β, and CCL3 to the same, or greater, extent as WT controls (Figure 1D).

**Figure 2:**
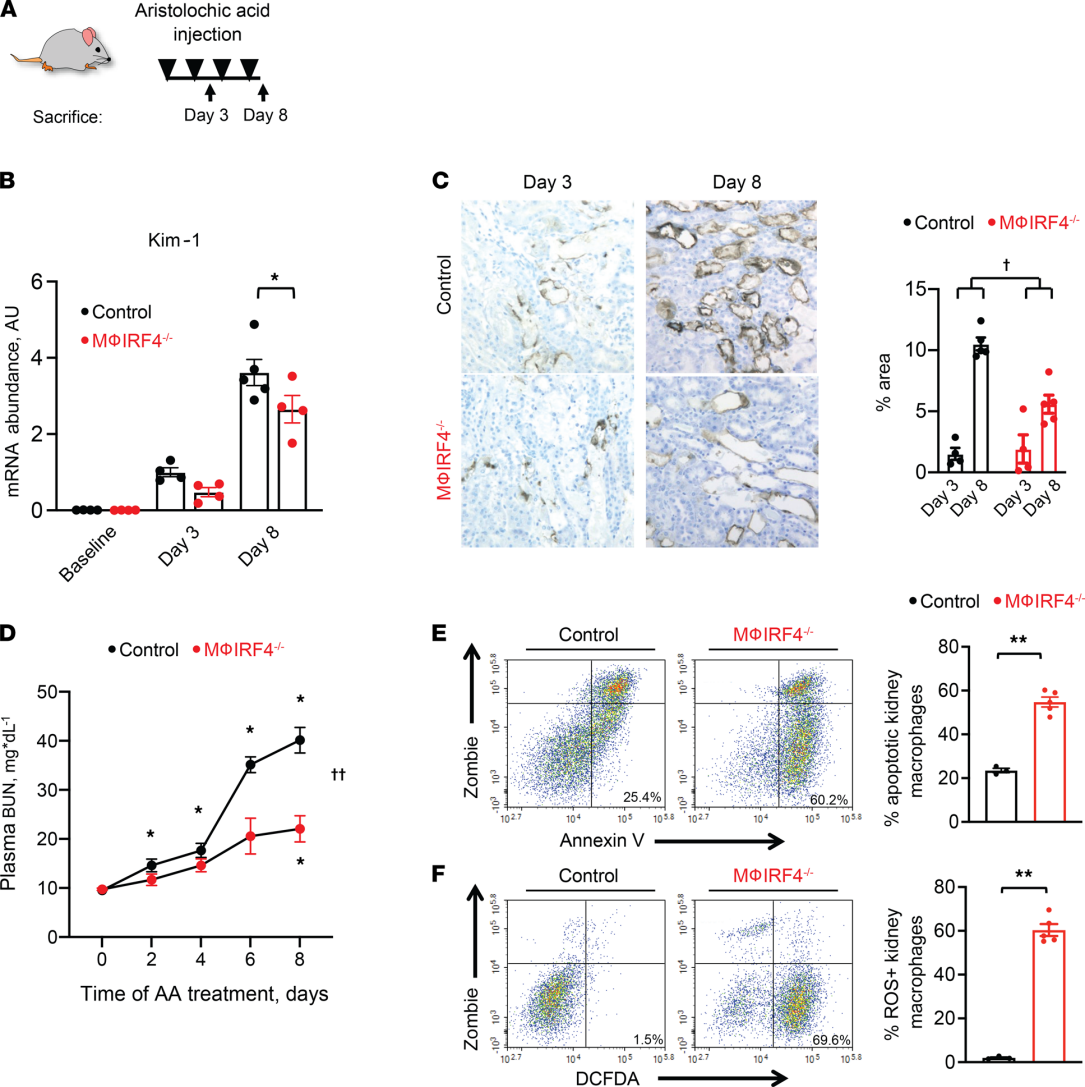
Effects of AA administration on renal function and kidney injury. (**A**) Experimental protocol. Animals received i.p. injections of AA (4mg/kg) every other day and were euthanized either at day 3 or day 8 after initial AA injection. (**B**) Total kidney mRNA abundance of Kim-1 at baseline and on days 3 and 8 of AA administration in WT control and macrophage IRF4^–/–^ mice. (**C**) Kim-1 renal epithelial protein expression on days 3 and 8 of the AA administration protocol in WT control and macrophage IRF4^–/–^ animals. Five fields of each kidney section were randomly selected, and the ratios of DAB-positive area to total areas in each field were counted in each high-power field using ImageJ software. (**D**) Plasma BUN following AA administration in WT and macrophage IRF4^–/–^ animals. (**E** and **F**) Effects of AA administration on kidney macrophage (**E**) apoptosis and (**F**) ROS production in WT control and macrophage IRF4^–/–^ animals on day 8 of the AA protocol. For **A** and **B**, *n* = 4 for baseline and day 3 time points for both genotypes; *n* = 5 for the day 8 time point for control mice and 4 for macrophage IRF4^–/–^ mice. For **C**, *n* = 4 per group for day 3 and 5 per group for day 8. For **D**, *n* = 5 per group. For **E** and **F**, *n* = 3 for WT control and 5 for macrophage IRF4^–/–^ mice. *P < 0.05 by 2-way ANOVA with repeated measures followed by Šidák post hoc test to compare within or between genotype differences at indicated time points. †*P* < 0.05 for interaction of genotype and treatment variables by 2-way ANOVA. ††*P* < 0.05 for interaction by 2-way ANOVA with repeated measures. **P < 0.05 by unpaired Student’s *t* test. Mϕ, macrophage. Note that all animals were treated with AA except baseline groups in **B**.

These results suggested that AA can directly stimulate a migratory and proinflammatory phenotype in macrophages. We next sought to determine if this phenomenon contributes to AA-mediated damage in vivo. To characterize acute effects in our model, we performed repeated injections of AA in WT control mice and monitored acute kidney injury at both the functional and histological levels (Figure 2A). Consistent with prior reports, AA caused acute proximal tubule damage, as demonstrated by increased epithelial expression and total kidney mRNA abundance of kidney injury molecule 1 (Kim-1) as early as 3 days into our protocol (Figure 2, B and C). Despite clear evidence of early epithelial injury, acute elevations in plasma blood urea nitrogen (BUN) concentrations were minimal at this time (Figure 2D). By 1 week into the protocol, however, we observed a more dramatic increase in epithelial Kim-1 abundance as well as plasma BUN (Figure 2, C and D).

The delayed injury response suggested a role for an inflammatory cell infiltration following initial epithelial damage. Our previous data demonstrated an important role for macrophage IRF4 in kidney infiltration and subsequent injury (7). As already demonstrated, IRF4 deletion reduced AA-induced macrophage migration, so we used macrophage IRF4^–/–^ animals to determine effects of macrophage-specific IRF4 deletion in our AA model. Compared with controls, macrophage IRF4^–/–^ mice were protected from acute elevations in epithelial Kim-1 as well as plasma BUN (Figure 2, B–D). Consistent with our findings in ex vivo macrophages, we observed higher levels of apoptosis and ROS production in renal macrophages from macrophage IRF4^–/–^ mice on day 8 of AA treatment, as assessed via flow cytometry (Figure 2, E and F, and Supplemental Figure 4A). Additionally, there were not detectable differences in cell cycle between genotypes (Supplemental Figure 4B).

**Figure 3:**
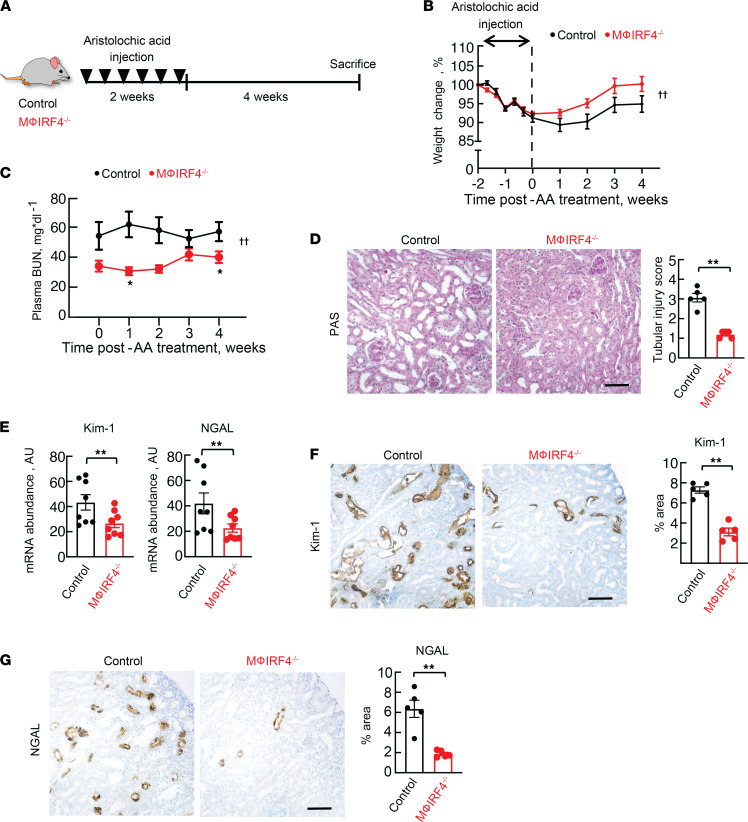
Effects of macrophage-specific IRF4 deletion on chronic kidney injury following AA administration. (**A**) Experimental protocol. Animals received i.p. injections of AA (4 mg/kg) every other day for 2 weeks. Mice were euthanized 4 weeks after the last dose of AA. Effects of AA on (**B**) body weight and (**C**) plasma BUN in control and macrophage IRF4^–/–^ animals throughout AA administration. Effects of AA on kidney injury, as measured by (**D**) tubular injury score, (**E**) total kidney Kim-1 and NGAL mRNA abundances, and protein abundance of (**F**) Kim-1 and (**G**) NGAL in control and macrophage IRF4^–/–^ animals. For **F** and **G**, 5 fields of each kidney section were randomly selected and the ratios of DAB-positive area to total areas in each field were counted in each power field using the ImageJ software. For **B** and **C**, *n* = 8. For **D**, **F**, and **G**, *n* = 5 per group. For **E**, *n* = 8 per group. ††*P* < 0.05 for interaction by 2-way ANOVA with repeated measures. *P < 0.05 by 2-way ANOVA with repeated measures followed by Šidák post hoc test to compare between genotype differences at indicated time points. **P < 0.05 by unpaired Student’s *t* test. AA, aristolochic acid; Mϕ, macrophage. Scale bar: 50 μM. Note that all animals were treated with AA.

We examined if protective effects of IRF4 deletion persisted into the chronic kidney injury phase by studying a different group of macrophage IRF4^–/–^ mice and controls 4 weeks after repeated AA injections (Figure 3A). Weight loss during AA treatment was not different between groups, as both lost 5%–10% of their baseline body weight (Figure 3B). In the period after treatment, knockout mice regained body weight and returned to their baseline weight faster than control animals. Plasma BUN was also lower in macrophage IRF4^–/–^ animals following AA treatment (Figure 3C). Injury protection was evident at the histological level, as shown by reduced tubular injury score and decreased total kidney abundance of Kim-1 and neutrophil gelatinase-associated lipocalin (NGAL) at the transcript and protein levels (Figure 3, D–G).

**Figure 4:**
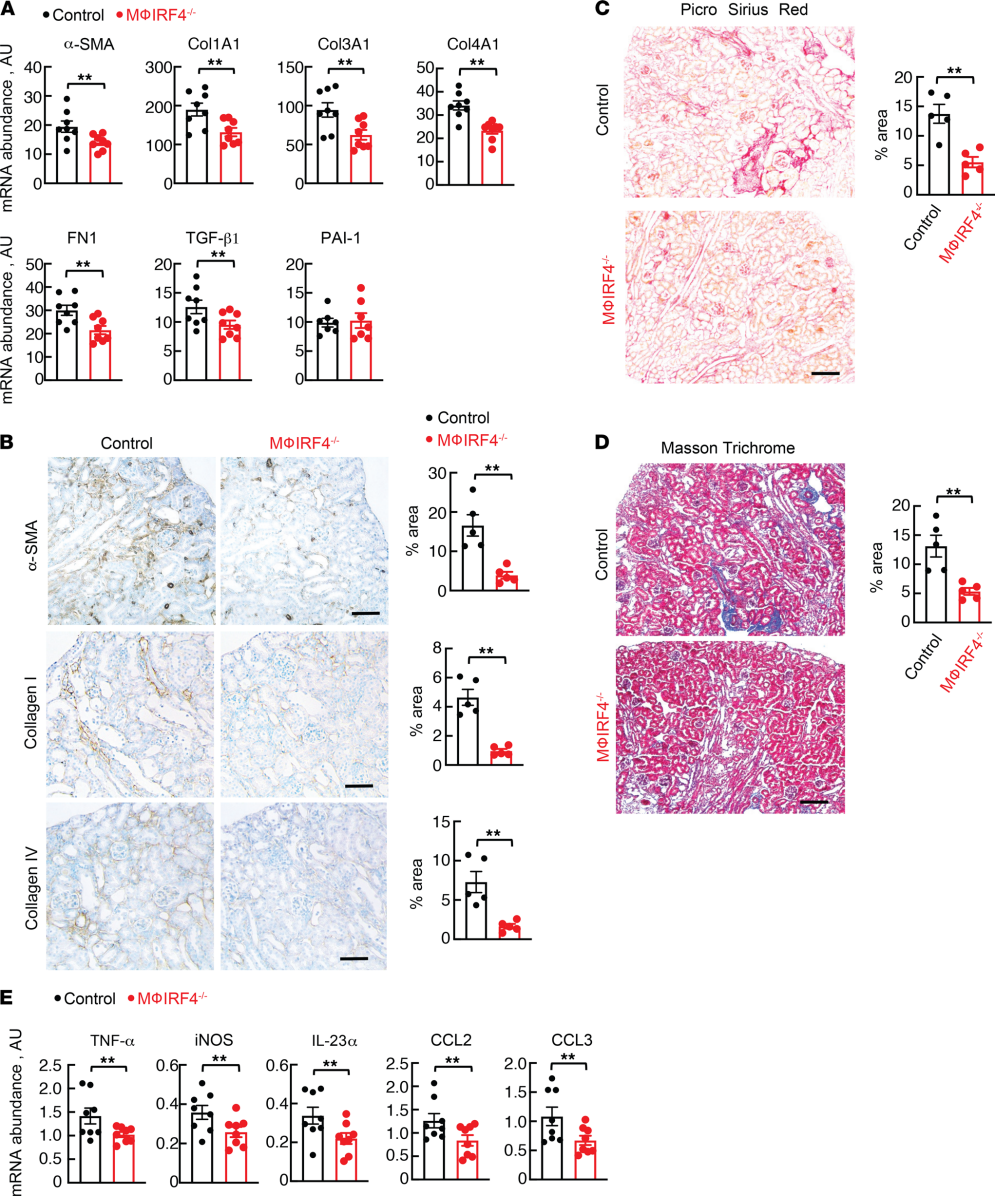
Effects of long-term AA administration on kidney fibrosis and inflammation. (**A**) Total kidney mRNA abundance of α-SMA, Col1A1, Col3A1, Col4A1, FN1, TGF-β1, and PAI-1 in control and macrophage IRF4^–/–^ mice following AA administration. (**B**) Protein abundance of α-SMA, collagen I, and collagen IV; (**C**) Picrosirius red; and (**D**) Masson’s trichrome staining following AA administration in control and macrophage IRF4^–/–^ animals. Five fields of each kidney section were randomly selected, and the ratios of (**B**) DAB-positive area, (**C**) Picrosirius red–positive area, or (**D**) blue area to total areas in each field were counted in each high-power field using ImageJ software. (**E**) Total kidney mRNA abundance of proinflammatory cytokines, including TNF-α, iNOS, IL-23α, CCL2, and CCL3, in control and macrophage IRF4^–/–^ mice following AA administration. For **A** and **E**, *n* = 8 per group. For **B–D**, *n* = 5 per group. **P < 0.05 by unpaired Student’s *t* test. AA, aristolochic acid; Mϕ, macrophage. Scale bar: 100 μM, except (**B**) where scale bar: 50 μM. Note that all animals were treated with AA.

In addition to reduced kidney injury, macrophage IRF4^–/–^ animals demonstrated decreased renal fibrosis at both the transcript and protein levels 4 weeks following AA treatment. There were reductions in total kidney transcript abundance of fibrotic and profibrotic factors, including α smooth muscle actin (αSMA), type 1 collagen (Col1A1), type 3 collagen (Col3A1), type 4 collagen (Col4A1), fibronectin (FN1), and TGF-β1, though differences were not observed for plasminogen activator inhibitor 1 (PAI-1) (Figure 4A). A similar trend of reduced fibrosis was seen at the protein level, with decreased abundance of αSMA, type 1 collagen, and type 4 collagen; reduction in fibrosis was confirmed by Picrosirius red and Masson’s trichrome staining (Figure 4, B–D). Consistent with injury protection, we detected reduced total kidney abundance of the proinflammatory cytokines TNF-α, inducible nitric oxide synthase (iNOS), IL-23α, CCL2, and CCL3 (Figure 4E).

**Figure 5:**
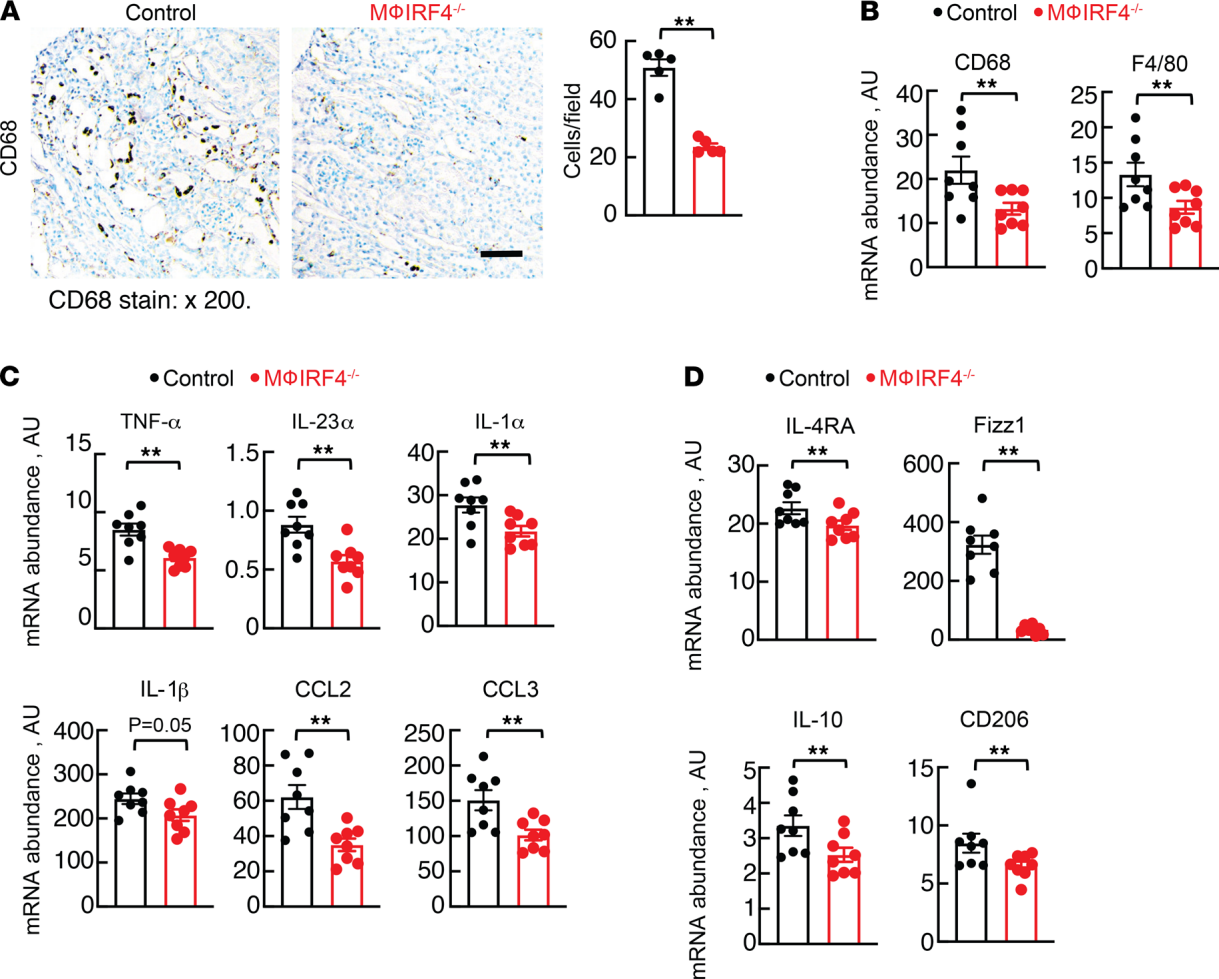
Chronic AA effects on kidney macrophage phenotype. (**A**) Renal CD68 protein abundance in control and macrophage IRF4^–/–^ animals following AA administration. (**B**) Total kidney CD68 and F4/80 mRNA abundance following AA administration. Five fields of each kidney section were randomly selected, and the total number of DAB-positive cells per field was counted in a blinded fashion. Effects of chronic AA administration on (**C**) proinflammatory (TNF-α, IL-23α, IL-1α, IL-1β, CCL2, and CCL3) and (**D**) proresolving (IL-4Rα, Fizz1, IL-10, and CD206) mRNA abundance in isolated macrophages from control and macrophage IRF4^–/–^ animals. For **A**, *n* = 5 per group. For **B–D**, *n* = 8 per group. **P < 0.05 by unpaired Student’s *t* test. AA, aristolochic acid; Mϕ, macrophage. Scale bar: 50 μM. Note that all animals were treated with AA.

To determine if reduced renal macrophage abundance in macrophage IRF4^–/–^ animals contributed to the improved renal function and decreased injury and fibrosis after long-term AA treatment, we quantified renal macrophages via CD68 immunostaining. We observed fewer macrophages in the knockout mice and also detected reduced total kidney mRNA abundance of the macrophage markers CD68 and F4/80 (Figure 5, A and B). There was also reduced abundance of CD3-positive T cells in knockout animals (Supplemental Figure 5). Isolated renal macrophages following long-term AA treatment showed alterations in macrophage phenotype, as proinflammatory markers, including TNF-α, IL-23α, IL-1α, IL-1β, CCL2, and CCL3, and proresolving markers, including IL-4 receptor α (IL-4Rα), found in inflammatory zone 1 (Fizz1), IL-10, and cluster of differentiation 206 (CD206) were decreased in macrophage IRF4^–/–^ animals compared with controls (Figure 5, C and D).

## Discussion

Macrophages are critical to both the propagation and resolution of kidney injury. Following damage to intrinsic kidney cells, infiltration of macrophages, along with other immune cell subsets, drives the injury response. Despite the key role of macrophages in the injury process, current thinking identifies them as being modulators of kidney injury (6). In this paradigm, kidney injury initially involves the kidney tissue itself, with the proximal tubule being a major target; the immune system, including macrophages, is then secondarily activated following the release of damage associated molecular patterns (DAMPs) and chemoattractants to modulate ongoing kidney injury. This important distinction between injury targets and secondary modulators of such injury explains why experimental manipulation of macrophage function can ameliorate injury but does not prevent it entirely. The current study revises this model to show that AA directly acts upon macrophages to promote migration and a proinflammatory phenotype ex vivo. Further, we have demonstrated that macrophage deletion of IRF4, a known regulator of macrophage function (7), ameliorates AA-induced kidney injury and subsequent chronic renal damage and fibrosis.

It is well-established that AA causes direct tubular injury, resulting in progressive interstitial fibrosis and renal failure in patients (8, 9). While the mechanisms through which this injury occurs are not entirely understood, it is clear that AA forms DNA adducts, resulting in oxidative stress (10), mitochondrial dysfunction (11), and subsequent apoptosis of renal tubular epithelial cells (9). This is accompanied by both fibroblast activation and inflammatory cell infiltration (12). While the direct effects of AA on macrophages described herein represent a unique example of toxin-induced stimulation driving immune cell–mediated injury, this is not the first description of the role of immune cells in AA-induced kidney injury. Baudoux et al. described the importance of T cell subsets in a model of AAN, showing that CD4^+^ or CD8^+^ T cell depletion resulted in more severe acute kidney injury following AA administration (13). Their T cell depletion protocols were accompanied by shifts in myeloid cell subpopulations that may have contributed to the observed phenotypes. While we observed reduced T cell abundance in our knockout model, it is unclear if this is merely a result of having less chronic kidney injury or if it actively mediates protection, possibly through crosstalk with myeloid cells. Honarpisheh et al. demonstrated an increase in macrophage number following AA treatment and described changes in macrophage surface markers and ROS production (14). They also observed AA-induced apoptosis in macrophages ex vivo. While we did not see this in our studies, the dose of AA we used was lower than theirs, which likely accounts for this difference. Our IRF4-knockout model specifically targets macrophages and resulted in reduced injury due to inhibition of macrophage migration to the injured kidney, highlighting the importance of the myeloid lineage in AA-induced kidney injury.

IRFs are a group of transcription factors (IRF1–IRF9) that mediate transcription of interferons and play an important role in regulation of the immune system. IRF4 is a well-described modulator of adaptive immunity and is necessary for maturation of both T and B cells (15), Treg function (16), and Th17 cell differentiation (17). In addition, it is classically described as an antiinflammatory mediator in macrophages and dendritic cells (18–21) and is critical for dendritic cell development (22). In vitro, IRF4 is known to mediate macrophage polarization to an M2 phenotype (23, 24). In the current study, IRF4 deletion altered direct AA effects on macrophages ex vivo by preventing AA-induced migration and enhancing rates of ROS production, yet it did not diminish the proinflammatory influence on macrophage phenotype. These results provide important insight into the role of macrophage IRF4 in AAN. They suggest that effects of IRF4 deletion on migration predominate in protecting macrophage IRF4^–/–^ animals in this study by preventing infiltration of proinflammatory macrophages. Our group recently reported that macrophage IRF4 deletion inhibits migration via reduced phosphoinositide 3-kinase/AKT signaling (7), and our current findings suggest that AA may increase migration via a similar mechanism.

Protective effects in macrophage IRF4^–/–^ animals were also associated with increased rates of macrophage apoptosis, which is consistent with prior reports of an antiapoptotic role of IRF4 (25). This finding suggests that even the myeloid cells that are able to infiltrate the kidney have higher rates of macrophage cell death, which could add another layer of protection. The increased rates of ROS production in macrophages lacking IRF4 may be secondary to the increased rates of apoptosis or may be associated with the AA-mediated polarization to an inflammatory phenotype. The equivalent, or enhanced, increase in inflammatory cytokine transcript abundance in knockout cells following AA treatment is consistent with the literature that shows that IRF4 negatively regulates proinflammatory cytokine production, and its deletion prevents macrophage polarization to an M2 phenotype (26). In sum, these results suggest that the observed protective effects were not through changes in macrophage phenotype but were due to decreased migration and increased apoptosis.

Our group has now reported that macrophage-specific IFR4-knockout mice are protected from acute and chronic kidney injury in 3 models: IRI, UUO, and AAN. Interestingly, reports using global IRF4-knockout animals demonstrated worsening of both acute and chronic kidney injury following IRI. As already stated, IRF4 is known to be extremely important in lymphoid cells, in addition to the myeloid lineage, and may play a role in other cells types as well. The differences between our cell-specific model and the global knockout highlight the pleiotropic effects of IRF4 in multiple cell types. While myeloid deletion is protective from kidney injury, on a global level its deletion appears deleterious. Future studies using cell-specific deletion of T and B cell subsets will permit a granular level of analysis delineating cell-specific functions of IRF4.

In conclusion, we have demonstrated that AA directly acts upon macrophages to induce a proinflammatory and promigratory phenotype. Deletion of IRF4 in macrophages prevents the migration effect ex vivo and protects animals from AA both acutely and chronically in vivo. These results revise our current model of macrophage biology in kidney injury and show that macrophages themselves are stimulated directly by a toxin in the pathogenesis of acute kidney injury. Furthermore, they highlight the importance of IRF4 in mediating kidney injury and identify it as a potential target for the future development of novel therapies for kidney disease.

## Methods

### Mice.

Both IRF4-floxed (IRF4^fl/fl^) mice (stock no. 009380) and LysM-Cre mice (stock no. 004781) were purchased from The Jackson laboratory. All mice were on a C57BL/6J background. The LysM-Cre mice were crossed with the IRF4^fl/fl^ mice, and the resultant LysM-Cre; IRF4^f/+^ mice were then crossed with IRF4^fl/fl^ mice to get IRF4^fl/fl^ (control) mice and LysM-Cre; IRF4^fl/fl^ (macrophage IRF4^–/–^) mice. Age-matched male littermates (8–12 weeks old) were used for the experiments. All mice were genotyped with PCR before and after experiments.

### AA model.

For chronic experiments, all mice received i.p. injections of AA (MilliporeSigma, 4.0 mg/kg per injection) every other day for 2 weeks for a total of 6 injections and were euthanized 4 weeks after the last injection unless stated otherwise. For acute experiments, animals received the same dose every other day for either 3 or 8 days, as indicated in the figures.

### Renal macrophage isolation.

Renal macrophages and dendritic cells were enriched using a mixture of mouse CD11b and CD11c Microbeads and MACS columns (Miltenyi Biotec) following the manufacturer’s protocol as previously reported (7).

### BUN measurements.

Blood was collected in heparinized tubes on the indicated days via tail vein. Blood was centrifuged for 5 minutes at 2000*g* using a 5415D tabletop centrifuge (Eppendorf), and the plasma layer was removed and stored at –20°C until measurements were performed. Serum BUN was measured using a Urea Assay Kit (BioAssay Systems) according to the manufacturer’s protocol.

### Immunohistochemistry.

Following euthanasia, kidneys were removed and incubated at room temperature overnight in fixative containing 3.7% formaldehyde, 10 mM sodium m-periodate, 40 mM phosphate buffer, and 1% acetic acid. The fixed kidney was dehydrated through a graded series of ethanols, embedded in paraffin, sectioned (5 μm), and mounted on glass slides. Immunostaining was carried out as in previous reports (7). Antibodies used include: anti-KIM1 (AF1817, R&D Systems), anti-NGAL (AF1857, R&D Systems), anti-CD68 (ab125212, Abcam), anti-αSMA (A5228, MilliporeSigma), anti–type 1 collagen (600-401-103-0.1, Rockland), and anti–type 4 collagen (600-401-106-0.1, Rockland). For calculation of percentage area, 5 fields on the kidney section were randomly selected, and the ratios of DAB-positive area to total areas in each field were counted in each power field using ImageJ software (NIH). For CD68 and CD3 staining, the number of positive cells was counted in a blinded fashion. Quantification was performed using ImageJ software as previously reported (27).

### Real time qPCR.

RNA from kidneys and isolated renal CD11b^+^CD11c^+^ cells was isolated using Trizol reagent (Invitrogen). The SuperScript IV First-Strand Synthesis System kit (Invitrogen) was used to synthesize cDNA from equal amounts of total RNA from each sample. Quantitative RT-qPCR was performed using TaqMan real-time PCR (7900HT, Applied Biosystems). Master Mix and all gene probes were purchased from Applied Biosystems. The probes used in the experiments included mouse TNF-α (Mm99999068), IL-23a (Mm00518984), IL-1α (Mm00439621), IL-1β (Mm00434228), CCL2 (MCP-1, Mm00441242), CCL3 (Mm00441258), Kim-1 (Havcr1, Mm00506686), NGAL (Lcn2, Mm01324470), α-SMA (Acta2, Mm01546133), Collagen I (col1a1, Mm00801666), Collagen III (col3a1, Mm01254476), Collagen IV (col4a1, Mm01210125), FN1 (Mm01256744), iNOS (Mm00440502), CD68 (Mm03047343), F4/80 (Emr1, Mm00802529), IL-6 (Mm00446190), GM-CSF (csf2, Mm01290062), IL-4Rα (Mm01275139), FIZZ1 (RELMα, Mm00445109), IL-10 (Mm01288386), CD206 (Mrc1, Mm01329362), IRF4 (Mm00516431), TGF-β1 (Mn01178820), PAI-1 (Serpine1, Mm01204470), RPS18 (Mn02601777), and Gapdh (Mm99999915). The amplification of specific PCR products was confirmed by the 2^(–ΔΔCT)^ method with dissociation curve analysis for each primer. Data were normalized to GAPDH or RPS18.

### Ex vivo macrophage culture.

Isolation of peritoneal macrophages was performed as previously described (7). Briefly, mice received an i.p. injection of 2 ml sterile thioglycollate medium (3% w/v of an autoclaved stock prepared from dehydrated thioglycollate medium and sterile saline water) (MilliporeSigma). Three days later, they received an i.p. injection of ice-cold PBS with 3% FBS. Peritoneal fluid was subsequently harvested and centrifuged, and pellets were resuspended in RPMI1640 medium supplemented with 100 U/ml penicillin, 100 μg/ml streptomycin, and 10% FBS and seeded in a 10 cm dish for 3 hours. After washing 3 times with culture medium, cells were counted using an automated cell counter (Bio-Rad, TC-20). The cells were then used for study. Cells were plated in 12-well plates (Corning) at a concentration of 1 × 10^6^ cells per well for flow cytometry studies or in 24-well plates (Corning) at a concentration of 2.5 × 10^5^ cells per well for qPCR studies. They were treated overnight with DMSO or AA (3.5 μM) prior to collection in Trizol reagent (Invitrogen) for qPCR analysis or H_2_DCFDA as described below. Cells were treated for 5 hours prior to annexin V analysis as described below.

### RAW 264.7 culture.

Cells were purchased from the ATCC and cultured in RPMI1640 supplemented with 10% FBS and 1% each penicillin and streptomycin. Cells were plated at a density of 5 × 10^5^ cells per well (12-well dish) prior culture with DMSO or 3.5 μM AA for 16 hours. Cells were harvested in Trizol reagent (Invitrogen) for qPCR analysis.

### Picrosirius red staining and Masson’s trichrome staining.

Picrosirius red staining (MilliporeSigma, 365548) and Masson’s trichrome staining (MilliporeSigma, HT15-1KT) was performed according to the protocol provided by the manufacturer. Quantification was performed using ImageJ software.

### Periodic acid–Schiff staining.

Staining was performed according to the protocol provided by the manufacturer. Briefly, slides were dewaxed, rehydrated, and treated with 1% periodic acid (MilliporeSigma, 395132) for 15 minutes. They were then rinsed in water, immersed in Schiff’s reagent (MilliporeSigma, 3952016) for 1 hour, rinsed in water, counterstained with Harris’s hematoxylin for 2 minutes, washed in running tap water, dehydrated, and mounted.

### Tubular injury score.

Analysis was performed by calculating the percentage of tubules at the corticomedullary junction that displayed cell necrosis, loss of the brush border, cast formation, and tubular dilatation: 0, none; 1, ≤10%; 2, 11%–25%; 3, 26%–45%; 4, 46%–75%; 5, >76%.

### Ex vivo macrophage migration assay.

The migration assay was performed as previously described (7). Freshly isolated macrophages (75,000 cells) were seeded in the top chamber of a 24-well PET membrane (8 μm pore size). Cells translocated to the lower chamber in response to exposure to DMSO or AA (3.5 μM) for 3 hours. Cells in the upper chamber were removed with a cotton swab, and the filters were fixed with 70% ethanol and stained with 0.2% crystal violet. Filters were photographed on a Leica DMi1 microscope, and total cell number was counted.

### Annexin V.

Staining was performed on peritoneal macrophages or digested kidney (as described below) according to the protocol provided by the manufacturer (Invitrogen, V13242). Cells were analyzed by flow cytometry as described below.

### ROS.

Staining was performed on peritoneal macrophages (as described above) or digested kidney (below) using H_2_DCFDA according to the protocol provided by the manufacturer (Invitrogen). Cells were analyzed by flow cytometry as described below.

### Flow cytometry.

Flow cytometry was performed as previously reported (7). Briefly, after perfusion of the kidneys with PBS, 1 kidney was removed, minced into fragments, and digested in RPMI 1640 containing 2 mg/ml collagenase type D and 100 μg/ml DNase I for 45 minutes at 37°C, with intermittent agitation. Kidney fragments were passed through a 40 μm mesh (Falcon; BD Biosciences), yielding single-cell suspensions. Cells were centrifuged (300*g*, 10 minutes, 4°C), resuspended in FACS buffer, kept on ice, and counted using a Bio-Rad TC20 automated cell counter. 10^5^ cells were incubated in 2.5 μg/ml Fc blocking solution and stained for 60 minutes at 4°C with antibodies, including FITC rat anti-mouse CD45, APC anti-Ly6G, PE/Cy7 anti-mouse F4/80, Pacific Blue anti-mouse CD11b, APC anti-mouse CD11c, or isotype control (all BioLegend). Cells were then washed and stained for 15 minutes at room temperature with annexin V or H_2_DCFDA and resuspended in 1× annexin binding buffer (or PBS with 1% BSA for H_2_DCFDA). For cell cycle analysis, cells were stained with propidium iodide at 50 μg/ml. After immunostaining, cells were analyzed immediately on a Novocyte flow cytometer with NovoExpress Software (Acea Biosciences) for data acquisition, and data analysis was performed using FlowJo v10 software (Tree Star).

### Statistics.

Data are shown as mean ± SEM. Comparisons over time were made with 1- or 2-way ANOVA with repeated measures followed by post hoc tests as indicated. Between group comparisons were made using 2-tailed Student’s *t* test or 2-way ANOVA with Šidák post hoc test as appropriate as indicated in figure legends. *P* < 0.05 was used as the significance threshold. Analysis was performed using Prism software.

### Study approval.

All animal experiments were performed in accordance with the guidelines of and with the approval of the Institutional Animal Care and Use Committee of Vanderbilt University Medical Center.

## Author contributions

KS, AST, RCH, and MZ conceived the study and designed experiments. KS, AST, AN, SW, YZ, JT, SC, SRB, and XF performed experiments. KS, AST, RCH, and MZ analyzed data. KS, AST, JPA, MZ, and RCH wrote and edited the manuscript.

## Supplementary Material

Supplemental data

## Version 1 01/13/2022

In-Press Preview

## Version 2 02/22/2022

Electronic publication
