# Supplementary material for: Macrophage interferon regulatory factor 4 deletion ameliorates aristolochic acid nephropathy via reduced migration and increased apoptosis
Source: JCI Insight. 2022 Feb 22;7(4):e150723. doi: 10.1172/jci.insight.150723 (PMC8876461; doi:10.1172/jci.insight.150723)

Supplemental Figure 1

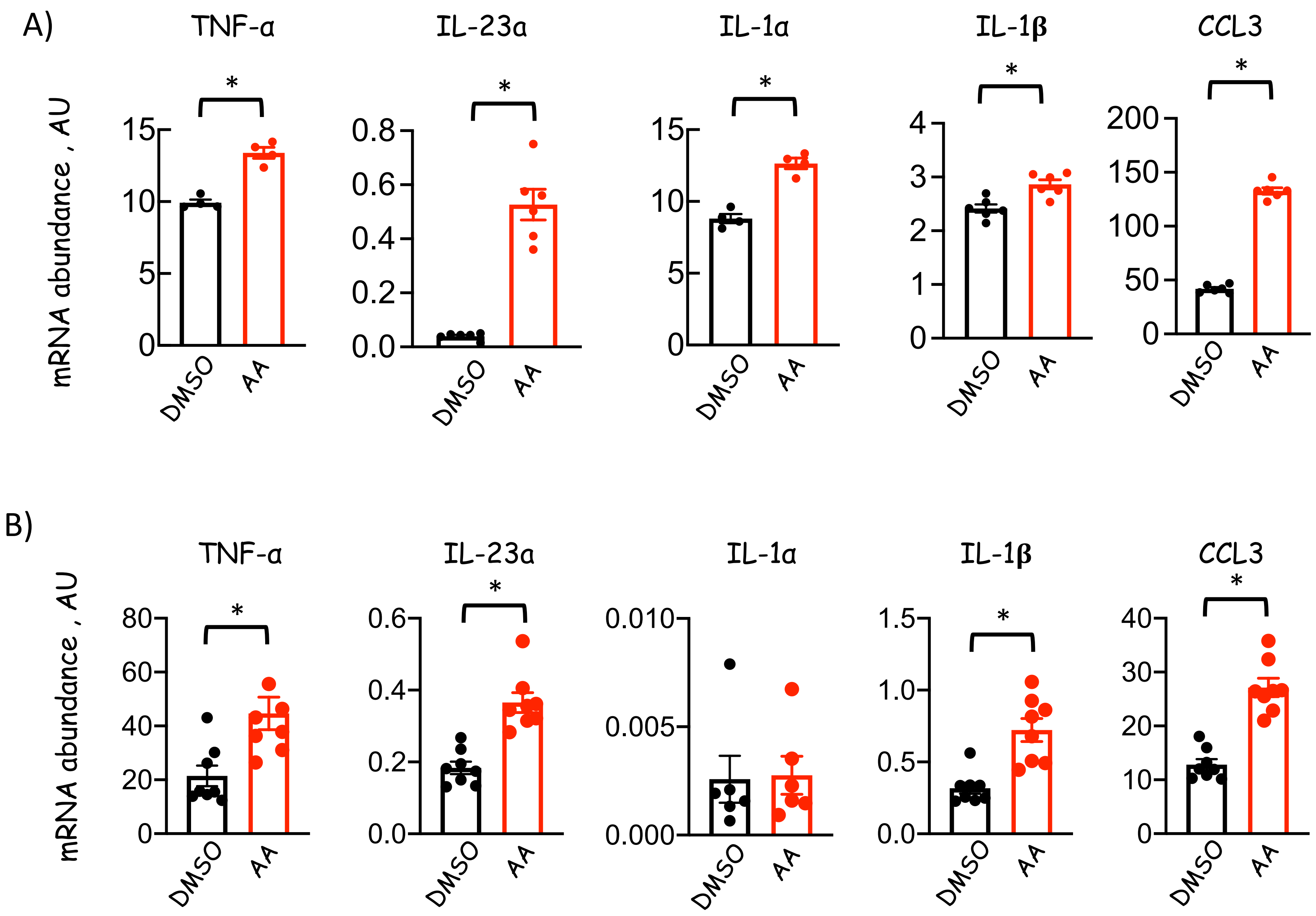

Supplemental Figure 2

IRF4 expression,
Renal macrophages

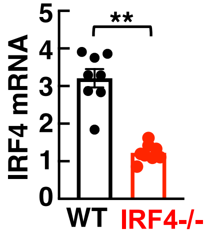

Supplemental Figure 3

A) Annexin V and ROS analysis, Peritoneal Macrophage + AA (3.5µM)

Gating strategy

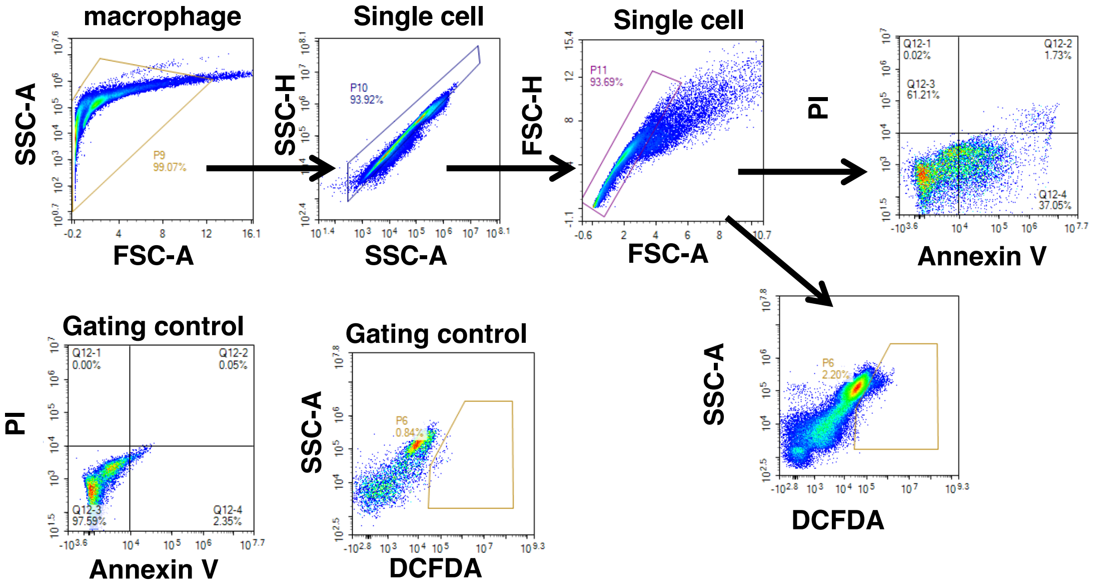

B)

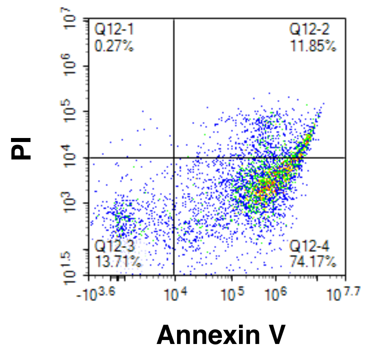

C)

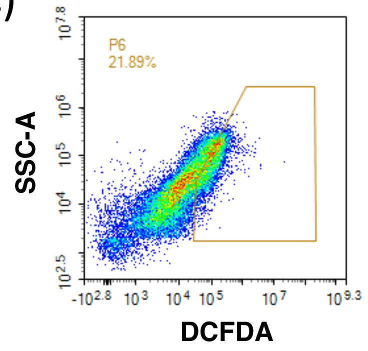

Supplemental Figure 4

A)

Gating strategy, Kidney tissue

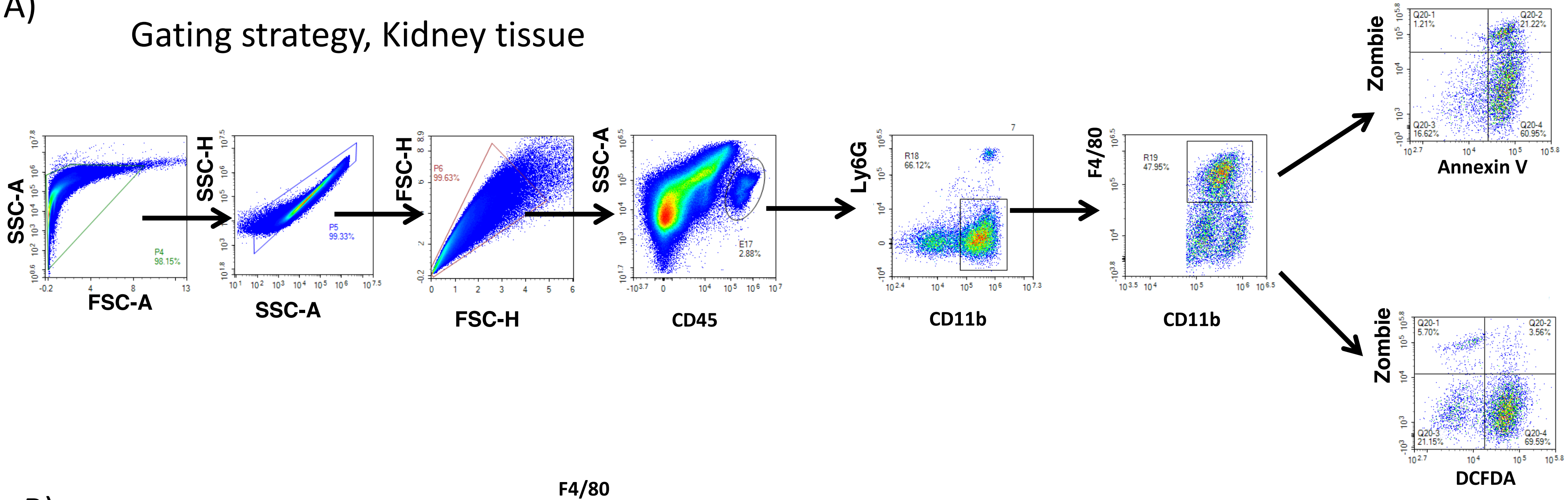

B)

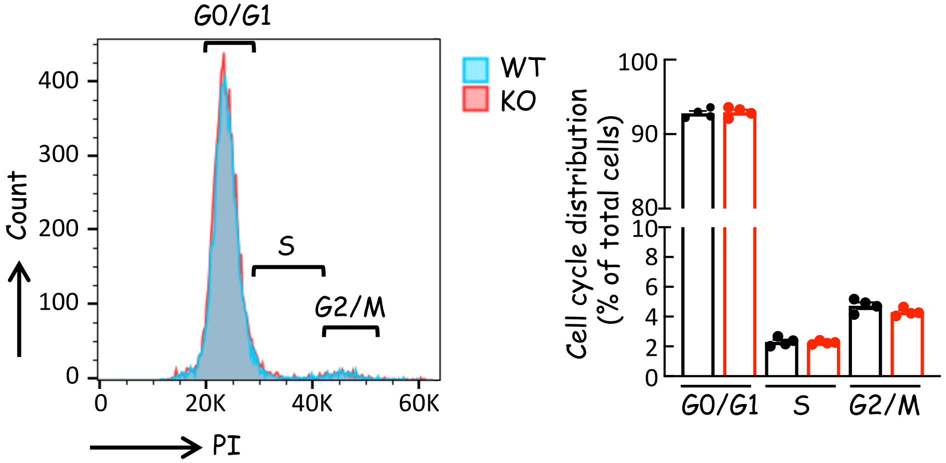

Supplemental Figure 5

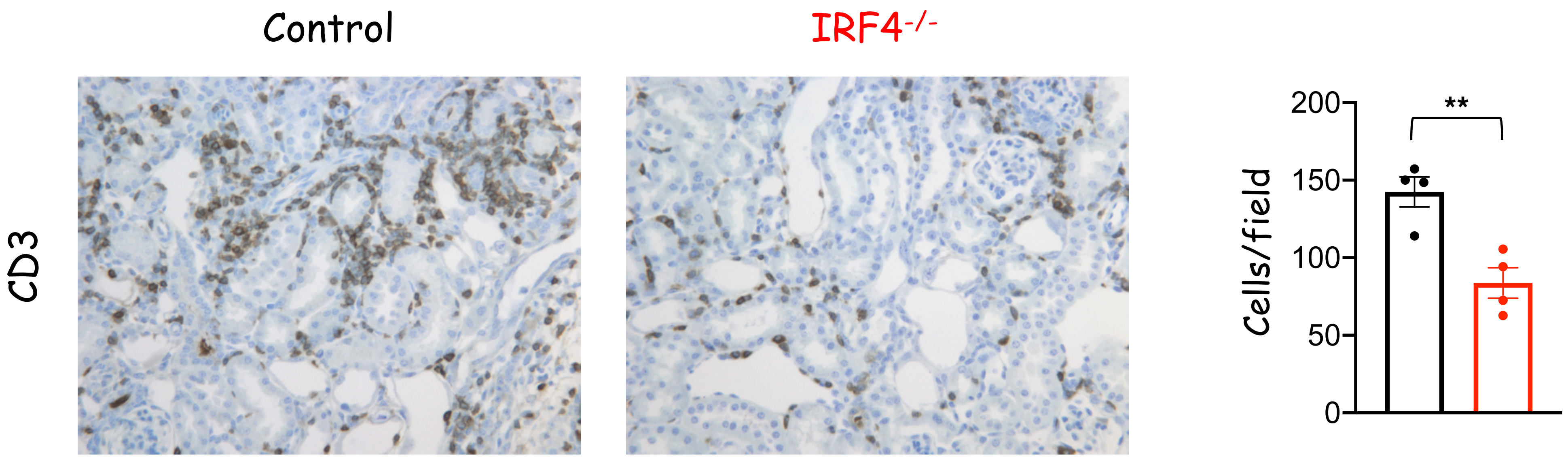

Supplement: Supplemental data [file jciinsight-7-150723-s099.pdf]
